# Percutaneous ALPITube ileostomy for colorectal anastomotic protection: a multicentre feasibility study

**DOI:** 10.1007/s10151-026-03330-8

**Published:** 2026-05-15

**Authors:** Carolina Germanetti, P. Mezzatesta, Tomás Elosua González, B. Defrancisco, B. Mussa

**Affiliations:** 1 Surgical Science Department, University of Turin, Città della Salute e della Scienza Hospital, Turin, Italy; 2 La Maddalena Oncological Center, Palermo, Italy; 3 Complejo Asistencial Universitario de León, Instituto de InvestigaciónBiosanitaria de León (IBIOLEÓN), León, España

**Keywords:** Protective ileostomy, Rectal surgery, Anastomotic protection, Minimally invasive surgery, Stoma complications, IDEAL framework, Surgical innovation

## Abstract

**Background:**

Standard loop ileostomy protects low colorectal anastomoses but causes substantial stoma-related morbidity including dehydration, high-output syndrome and metabolic complications. The Anastomotic Leak Prevention Ileostomy Tube (ALPITube) is a novel CE-marked percutaneous diversion device designed to provide anastomotic protection while avoiding traditional stoma complications.

**Objective:**

To evaluate the technical feasibility, safety profile and preliminary outcomes of ALPITube in an idea, development, exploration, assessment and long-term study (IDEAL) framework Stage 2a development study, with contextual comparison to published loop ileostomy benchmarks.

**Methods:**

Multicentre retrospective cohort study of consecutive patients undergoing elective colorectal surgery with low anastomosis and ALPITube diversion at two European centres – Clinica La Maddalena in Palermo, Italy (*n* = 28), and Hospital HM Regla in León, Spain (*n* = 15) – between May 2023 and June 2025. Primary outcome was 30-day complication rate. Secondary outcomes included device-specific adverse events, conversion to standard ileostomy and mortality.

**Results:**

Forty-three patients were analysed (mean age 69.7 ± 9.0 years; 88.4% with ≥ 1 comorbidity; 76.7% received neoadjuvant therapy). Device implantation was technically successful in all cases (mean implantation time 38 ± 14 min). Early complications occurred in 21 patients (48.8%), and were predominantly low-grade (Clavien–Dindo I–II: 25.6%). In total, five patients (11.6%) required conversion to loop ileostomy due to device maintenance difficulties. No anastomotic leaks or high-output syndrome occurred. No adverse events were associated with device removal. Contextual comparison with literature benchmarks suggested lower rates of high-output stoma (0% versus 16%), exit-site skin complications (18.6% versus 43%), postoperative ileus (4.7% versus 33%) and parastomal hernia (0% versus 8%).

**Conclusions:**

ALPITube demonstrates technical feasibility with an acceptable safety profile and signals potential reduction in stoma-specific complications. The 11.6% conversion rate reflects early implementation experience. These IDEAL Stage 2a findings support progression to prospective comparative studies.

**Supplementary Information:**

The online version contains supplementary material available at 10.1007/s10151-026-03330-8.

## Introduction

Protective diversion remains standard care for high-risk colorectal anastomoses, particularly following neoadjuvant therapy or ultra-low anterior resection. While effective in reducing the clinical impact of anastomotic leakage, loop ileostomy carries substantial morbidity affecting 21–70% of patients, with particular concern for metabolic complications that can compromise oncological care [1].

High-output stoma, defined as effluent exceeding 1500 mL per 24 h (or > 1000–1200 mL across two consecutive days), occurs in approximately 16% of patients, with dehydration accounting for 40–62% of ileostomy-related readmissions [2, 3]. Acute kidney injury develops in 22–26% of patients, often requiring emergency hospitalization [4, 5]. These metabolic disturbances create a cascade of complications including malnutrition and electrolyte imbalances that persist for months. For patients with colorectal cancer requiring adjuvant chemotherapy, these complications frequently necessitate treatment delays or dose reductions, potentially compromising oncological outcomes [6, 7]. The two-stage paradigm of stoma creation and subsequent closure compounds these challenges, with closure-related morbidity affecting 17–28% of patients [8].

Alternative strategies to mitigate ileostomy morbidity have been explored, including high-vigilance protocols with selective ileostomy construction guided by early inflammatory markers (as advocated by the Amsterdam group), very early closure within 7 days of index surgery (Bordeaux approach) and the “ghost ileostomy” technique using a silicone vessel loop around the terminal ileum exteriorized through the skin [9–11]. Each approach has limitations: selective protocols require robust surveillance infrastructure, early closure carries perioperative risk and ghost ileostomy still necessitates laparotomy if activated.

**Fig. 1:**
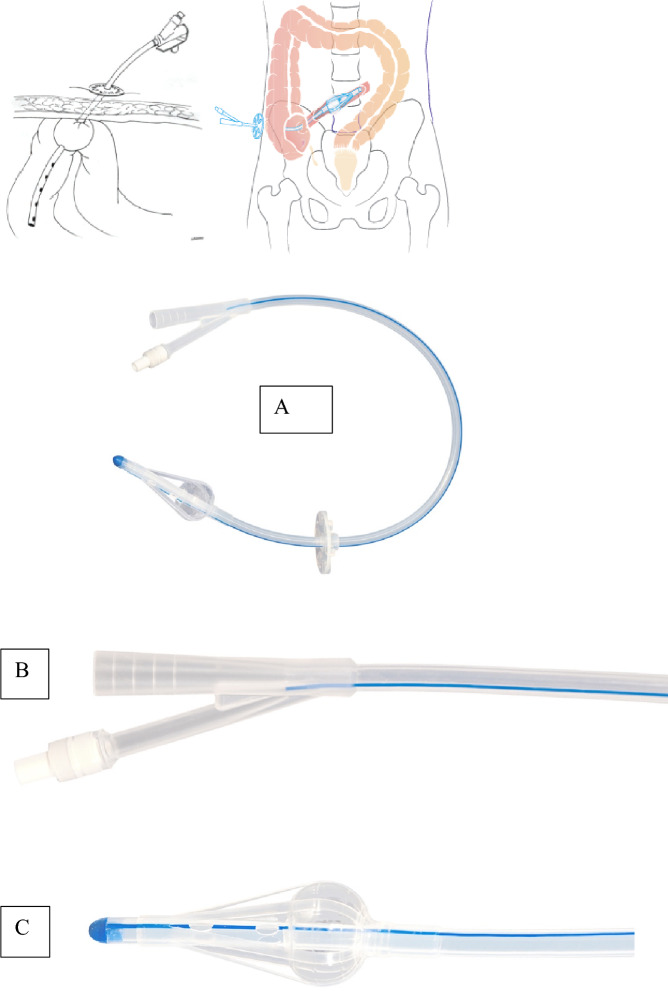
ALPITube device. **A** Complete device showing 40 cm silicone catheter with retention balloon (deflated), cage-protected drainage eyelets, Luer-lock inflation port, external retention plate and 15 mm connector for faecal collection bag. **B** Close-up of distal catheter showing the four drainage eyelets enclosed within the protective cage system proximal to the balloon. **C** Balloon inflated with 10 mL saline demonstrating low-pressure profile

Anastomotic Leak Prevention Ileostomy Tube (ALPITube) represents a novel approach to protective diversion, employing a percutaneous catheter system to achieve faecal stream diversion without creating a traditional stoma. The device is a CE-marked single-use medical device (Class IIa, certified under Directive 93/42/CEE and subsequent amendments; SIDAM s.r.l., Mirandola, Italy). It consists of a flexible medical-grade silicone catheter (40 cm length, 10 Fr outer diameter) equipped with a semi-rigid insertion mandrel, a low-pressure inflatable balloon (maximum 20 mL sterile saline) and four drainage eyelets protected by a cage system that prevents mucosal occlusion of the apertures and permits vacuum-assisted aspiration of enteric contents when required (Fig. 1). An external retention plate anchors the device to the skin and prevents inadvertent inward migration. When positioned 30–40 cm proximal to the ileocecal valve, the inflated balloon occludes the ileal lumen and diverts intestinal contents externally through the drainage eyelets, thereby reducing mechanical, enzymatic and bacterial load on the colorectal anastomosis during the critical healing phase. The controlled-calibre drainage system prevents the unrestricted high-volume losses characteristic of traditional ileostomies while maintaining low intraluminal pressure (< 15 cm H_2_O). Per manufacturer specifications, the device must not be inflated with air or contrast medium (sterile saline only), and the maximum approved in situ duration is 29 days.

This study represents an idea, development, exploration, assessment and long-term study (IDEAL) framework Stage 2a (Development) evaluation [12] of ALPITube, assessing technical feasibility, the safety profile and preliminary outcomes in a real-world colorectal surgery population. Prior preclinical development established device safety in bench testing, and the first-in-human cases confirmed technical implantability. The present multicentre experience provides essential data on procedural refinement, complication patterns and outcome signals to inform progression towards prospective comparative trials (IDEAL Stage 2b/3).

## Methods

### Study design and setting

This multicentre retrospective cohort study analysed consecutive adults undergoing elective colorectal surgery with low anastomosis and ALPITube diversion at two European centres between 22 May 2023 and 30 June 2025 – Clinica La Maddalena in Palermo, Italy (28 patients, 65.1%) and Hospital HM Regla in León, Spain (15 patients, 34.9%). The study was conducted and reported in accordance with the Strengthening the Reporting of Observational Studies in Epidemiology (STROBE) guidelines for observational studies and the IDEAL framework for surgical innovation (Stage 2a: Development) [12].

### Patient selection

**Fig. 2:**
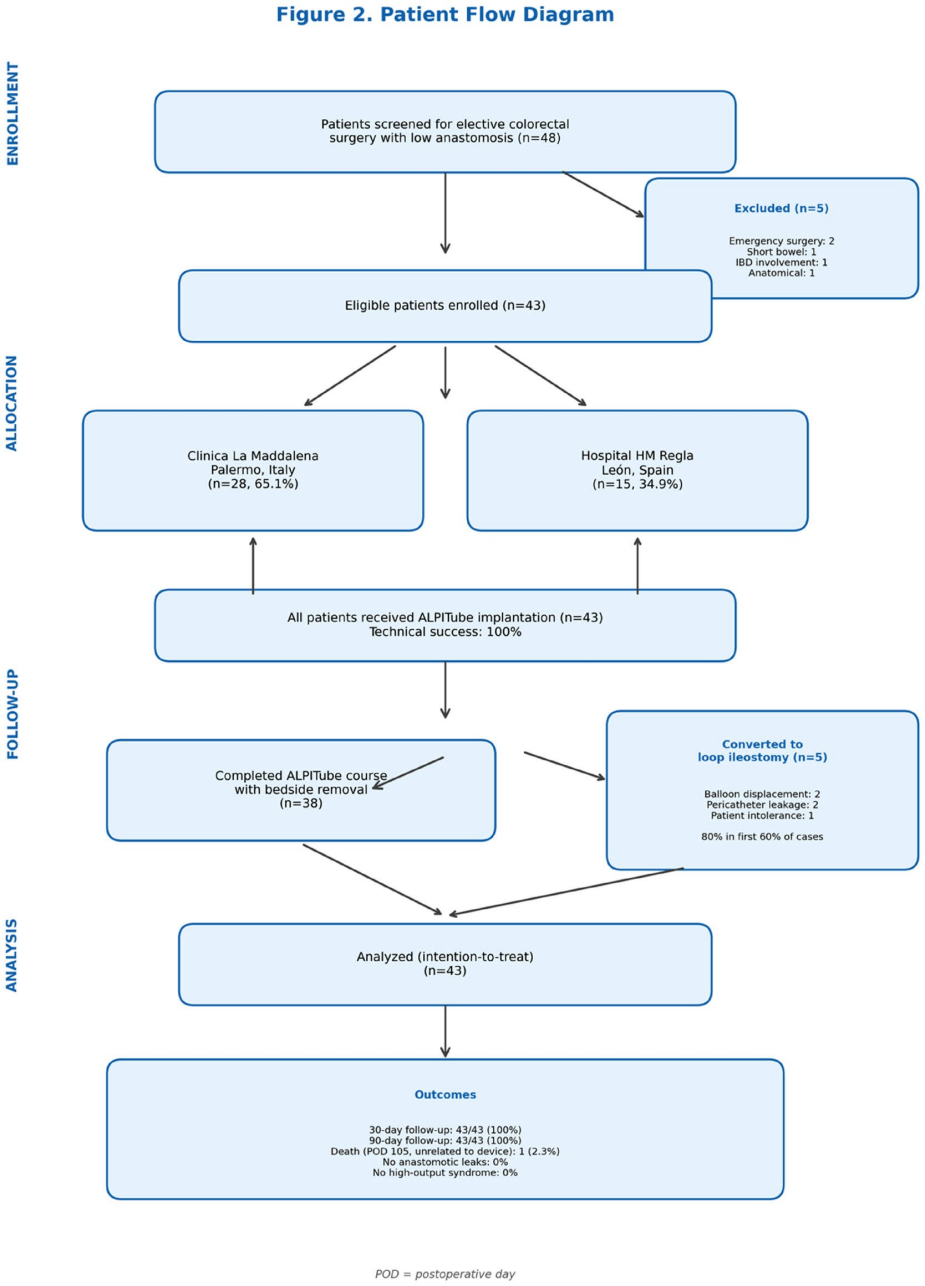
Patient flow diagram. STROBE flow diagram showing patient screening, eligibility assessment, inclusion across two centres (Palermo *n* = 28, León *n* = 15) and follow-up. Total of 43 patients analysed, 5 (11.6%) converted to loop ileostomy, 38 completed intended ALPITube course with bedside removal, and 1 death at day 105 unrelated to device

Inclusion criteria comprised adults (≥ 18 years) undergoing elective colorectal surgery with extraperitoneal colorectal anastomosis deemed to require protective diversion by the operating surgeon. Exclusion criteria included emergency surgery, short bowel syndrome (residual small bowel < 200 cm), inflammatory bowel disease with small bowel involvement and unsuitable anatomy for device placement. Patient flow is shown in Fig. 2.

### ALPITube system and implantation technique

The ALPITube system (SIDAM s.r.l., Mirandola, Italy; Ref. 49210169) consists of a 40 cm flexible silicone catheter (10 Fr outer diameter) incorporating a semi-rigid insertion mandrel, a low-pressure retention balloon (maximum inflation volume: 20 mL sterile saline) and four drainage eyelets protected by a cage system that maintains eyelet patency against mucosal contact and permits low-pressure vacuum-assisted aspiration (negative pressure 5–10 mmHg) when gravity drainage is insufficient. An external retention plate anchors the catheter to the skin, preventing proximal migration. The device connects to standard high-capacity faecal collection bags via a stepped 15 mm Luer-lock connector. Per instructions for use (IFU) specifications, the balloon must never be inflated with air or contrast medium (sterile saline only), and the maximum approved in situ duration is 29 days. All devices used in this study were employed within these manufacturer-specified parameters.

**Fig. 3:**
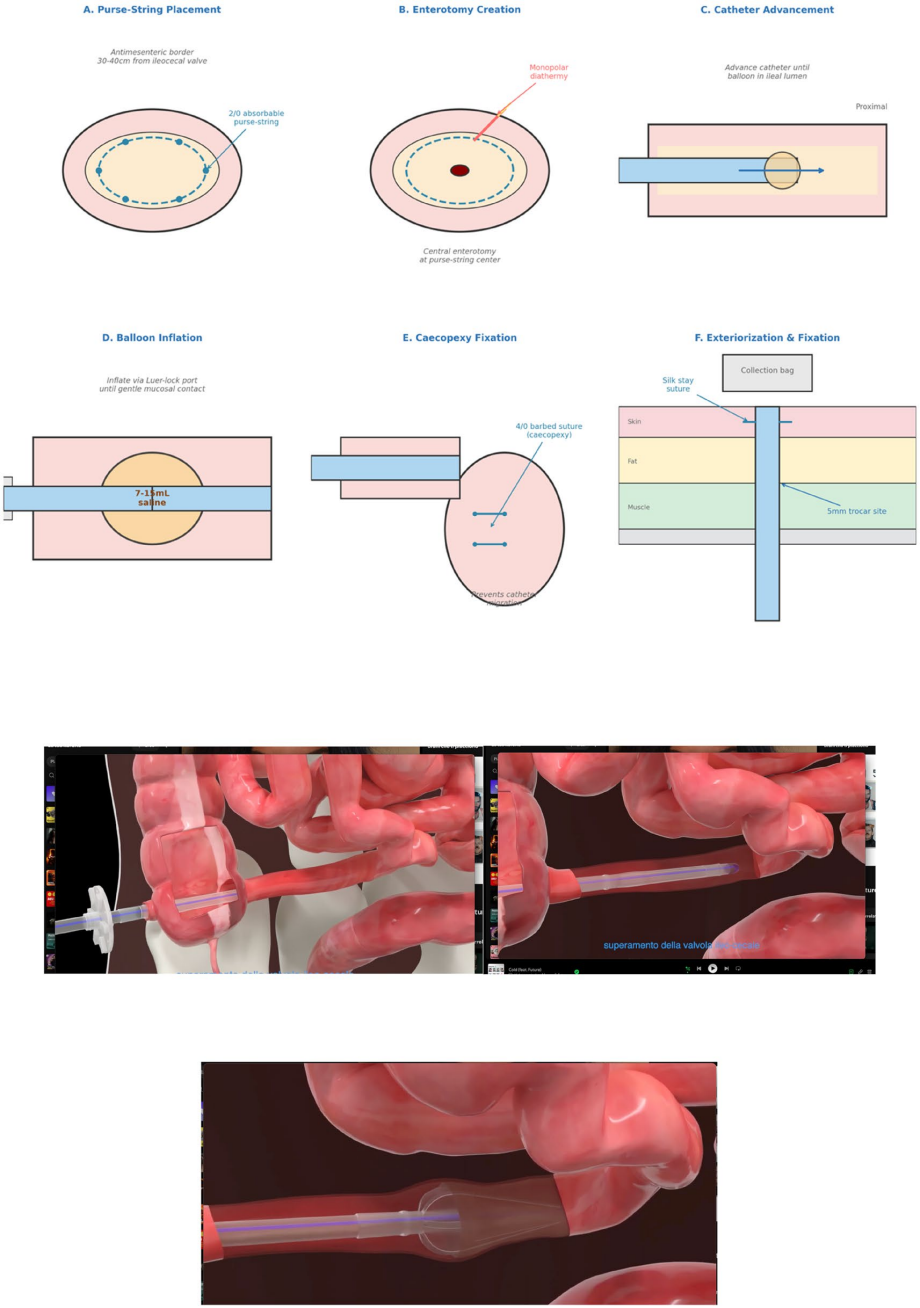
ALPITube implantation technique (step by step). **A** Purse-string suture placement on antimesenteric ileal border, 30–40 cm proximal to ileocecal valve. **B** Enterotomy creation at the centre of purse string. **C** Catheter advancement with balloon positioned within the ileal lumen. **D** Balloon inflation and purse-string tightening. **E** Caecopexy fixation to prevent migration. **F** Catheter exteriorization through 5 mm trocar site and skin fixation (QR code for supplementary video; Supplementary Material 1)

The implantation technique is presented in Fig. 3. Following completion of the primary colorectal anastomosis, a purse-string suture is placed on the antimesenteric border of the distal ileum, 30–40 cm proximal to the ileocecal valve (the manufacturer IFU specifies a 3/0 braided fast-absorbable suture; in this series, 2/0 absorbable suture was used per centre-specific protocol). An enterotomy is created at the centre of the purse-string using monopolar diathermy. The semi-rigid mandrel is fully inserted into the catheter lumen and the distal end lubricated with a water-soluble lubricant; the ALPITube catheter is then advanced proximally until the retention balloon lies entirely within the ileal lumen. The mandrel is removed, and the balloon is inflated with 7–15 mL sterile normal saline (mean 9.2 mL in this series; IFU maximum 20 mL) under direct laparoscopic vision until it gently apposes the mucosa without causing ischaemic blanching (air and contrast medium must not be used per IFU). The purse-string is tightened in a tobacco-pouch fashion using 3/0 absorbable suture, and the catheter is anchored to the caecal serosa (caecopexy) to prevent migration. The catheter is exteriorized through a 5 mm laparoscopic trocar site (avoiding creation of a new fascial defect), and the external retention plate is sutured to the skin, providing additional security against inward migration. A high-capacity faecal drainage bag is connected immediately.

### Postoperative management protocol

Patients received a low-residue diet from postoperative day 2, progressing to normal diet as tolerated. Daily effluent volumes were monitored and recorded. Per manufacturer IFU specifications, the main catheter lumen was flushed with an appropriate volume of sterile saline at least twice daily to maintain patency and prevent encrustation; volume and timing were documented in the clinical record. In cases where gravity drainage was insufficient, low-pressure vacuum-assisted aspiration was applied via the cage-protected eyelets at 5–10 mmHg (not exceeding 10 mmHg) for a limited duration until flow was re-established. The exit site was inspected daily and cleaned with tepid water and neutral soap using a circular motion outward from the catheter, and the skin was assessed for erythema, irritation or peri-catheter leakage. All patients remained hospitalized until anastomotic integrity was confirmed. On postoperative day 8 ± 1, computed tomography (CT) with trans-anal water-soluble contrast enema was performed to assess anastomotic integrity and identify any pelvic collections or subclinical leakage. Following confirmed anastomotic integrity, device removal was performed at the bedside: an empty syringe was connected to the Luer-lock valve port and the balloon deflated by aspiration, verifying that the volume aspirated matched the volume injected at implantation. The retention plate anchoring sutures were removed, and the catheter was withdrawn with gentle traction. The small enterotomy site (approximately 3–4 mm) was allowed to close spontaneously; no cases required surgical closure. A sterile colostomy bag was applied over the exit site for 24–48 h and then removed once spontaneous closure was confirmed.

### Outcome definitions

The primary outcome was 30-day complication rate using Clavien–Dindo classification. Secondary outcomes included:

- *Device-related adverse events*: balloon displacement (migration of balloon from intended position requiring repositioning or conversion), persistent peri-catheter leakage (ongoing leakage around catheter exit site despite conservative measures for > 48 h), device intolerance (patient-reported discomfort or distress prompting early removal or conversion) and removal-related complications (any adverse event occurring during or within 24 hours of device removal).
- *Conversion to standard loop ileostomy*: requirement for formal diverting ileostomy construction due to inability to maintain device function, documented with timing and indication.
- *High-output syndrome*: defined as effluent > 1500 mL/24 h or > 1200 mL on two consecutive days, consistent with established definitions [2].
- *Exit-site skin complications*: any peri-catheter skin injury including erythema, maceration, infection, or breakdown (replacing “peristomal” terminology as ALPITube does not create a formal stoma).
- *Postoperative ileus*: defined as functional inhibition of coordinated bowel motility without mechanical cause, evidenced by absent passage of flatus/stool, abdominal distension and intolerance to oral intake persisting beyond postoperative day 4.
- *Small-bowel obstruction*: defined as mechanical obstruction evidenced by imaging findings (dilated proximal loops with transition point) requiring active intervention.
- *Metabolic complications*: dehydration requiring intravenous fluid administration, acute kidney injury (increase in serum creatinine ≥ 0.3 mg/dL or ≥ 1.5× baseline) and clinically significant electrolyte abnormalities requiring correction.

Patients underwent clinical assessment at discharge, 30 days postoperatively and 90 days postoperatively. Follow-up data were available for all patients.

### Statistical analysis

Categorical variables were described using frequencies and percentages and continuous variables using means with standard deviations or medians with interquartile ranges. To provide clinical context, we performed one-sample binomial proportion tests comparing observed ALPITube complication rates with literature-derived benchmarks for loop ileostomy. These comparisons are presented as contextual information rather than formal comparative effectiveness analyses, given the absence of a concurrent control group and potential for confounding from differences in patient populations and outcome definitions. Statistical significance was set at *p* < 0.05. All analyses were performed using R statistical software (version 4.3.0).

## Results

### Patient characteristics

In all, 43 patients were analysed across two centres (Palermo: 28 patients, 65.1%; León: 15 patients, 34.9%). Baseline characteristics are presented in Table 1. The cohort was balanced for sex (51.2% male), with a mean age of 69.7 ± 9.0 years and mean body mass index (BMI) of 25.2 ± 4.2 kg/m^2^. Most patients (88.4%) had at least one comorbidity, reflecting a typical high-risk rectal cancer population. Colorectal cancer was the indication in 86% of patients, and neoadjuvant therapy was administered to 76.7%. This comorbidity-rich, neoadjuvant-exposed population represents a conservative test of device feasibility.

**Table 1:** Baseline patient characteristics (*n* = 43)

Characteristic	Value
Centre	
Clinica La Maddalena, Palermo, Italy	28 (65.1%)
Hospital HM Regla, León, Spain	15 (34.9%)
Male sex	22 (51.2%)
Age, years (mean ± SD)	69.7 ± 9.0
BMI, kg/m^2^ (mean ± SD)	25.2 ± 4.2
≥ 1 comorbidity	38 (88.4%)
Arterial hypertension	20 (46.5%)
Diabetes mellitus type II	7 (16.3%)
Previous cardiovascular event	6 (14.0%)
Prior abdominal surgery	9 (20.9%)
Colorectal cancer	37 (86.0%)
Neoadjuvant therapy (any)	33 (76.7%)
Chemotherapy	28 (65.1%)
Radiotherapy	31 (72.1%)
Tumour height from anal verge, cm (mean ± SD)^*^	9.0 ± 2.8

*BMI*  body mass index, *SD*  standard deviation

^*^Data available for 39 patients

### Operative and device parameters

Index procedures comprised anterior rectal resection (55.8%), low anterior resection (25.6%), ultra-low anterior resection (9.3%), Hartmann reversal (7.0%) and sigmoidectomy (2.3%). Mean total operative time was 265 ± 78 min. ALPITube implantation was technically successful in all 43 patients, requiring mean additional operative time of 38 ± 14 min (range 15–100). Mean balloon inflation volume was 9.2 ± 1.2 mL. The device remained in situ for a mean of 8.8 ± 2.0 days. Mean postoperative length of stay was 12.3 ± 5.6 days (Table 2).

**Table 2:** Operative profile and device parameters

Parameter	Value
Index procedure	
Anterior rectal resection	24 (55.8%)
Low anterior resection	11 (25.6%)
Ultra-low anterior resection	4 (9.3%)
Hartmann reversal	3 (7.0%)
Other (sigmoidectomy)	1 (2.3%)
Total operative time, minutes (mean ± SD)	265 ± 78
Device implantation time, minutes (mean ± SD)	38 ± 14
Balloon volume, mL (mean ± SD)	9.2 ± 1.2
Device in situ duration, days (mean ± SD)	8.8 ± 2.0
Postoperative length of stay, days (mean ± SD)	12.3 ± 5.6

*SD*  standard deviation

### Clinical outcomes and complications

Early complications (≤ 30 days) occurred in 21 patients (48.8%), with severity distribution shown in Table 3. The majority were low-grade: Clavien–Dindo Grade I in five patients (11.6%), Grade II in six (14.0%) and Grade III in eight (18.6%). No Grade IV or V complications occurred (excluding the single mortality). The most frequent complication was wound infection (18.6%). Late complications (31–180 days) occurred in two patients (4.7%).

**Table 3:** Postoperative complications and device-related events

Outcome	*n* (%)
Early complications (≤ 30 days)	21 (48.8%)
Clavien–Dindo classification	
Grade 0 (no complication)	22 (51.2%)
Grade I	5 (11.6%)
Grade II	6 (14.0%)
Grade III	8 (18.6%)
Grade IV	0 (0%)
Grade V (death)	1 (2.3%)
Major complications (Clavien–Dindo ≥ III)	8 (18.6%)
Specific complications	
Wound/exit-site infection	8 (18.6%)
Postoperative ileus	2 (4.7%)
Small bowel perforation	2 (4.7%)
Small bowel obstruction	1 (2.3%)
Left colon ischemia	1 (2.3%)
Device-related events leading to conversion	
Recurrent balloon displacement	2 (4.7%)
Persistent peri-catheter leakage	2 (4.7%)
Patient intolerance/discomfort	1 (2.3%)
Total conversions to loop ileostomy	5 (11.6%)
Events absent in cohort	
High-output syndrome	0 (0%)
Dehydration requiring admission	0 (0%)
Acute kidney injury	0 (0%)
Anastomotic leak	0 (0%)
Parastomal hernia	0 (0%)
Stenosis	0 (0%)
Superficial necrosis	0 (0%)
Removal-related adverse events	0 (0%)
Late complications (31–180 days)	2 (4.7%)
30-day mortality	0 (0%)
Overall mortality (at follow-up)	1 (2.3%)

### Device-related events and conversions

Five patients (11.6%) required conversion to standard loop ileostomy due to difficulties maintaining device position and function. The indications were recurrent balloon displacement requiring repositioning (*n* = 2), persistent peri-catheter leakage not resolved with conservative measures (*n* = 2, occurring before device removal during hospitalization) and patient intolerance related to discomfort (*n* = 1). Temporal analysis revealed that four of five conversions (80%) occurred in the first 60% of each centre’s case series, suggesting learning curve influence. All conversions were performed electively without emergent indications. The two cases of persistent leakage occurred during the in-hospital period prior to planned device removal and prompted conversion due to inadequate diversion function rather than exit-site complications.

**Fig. 4:**
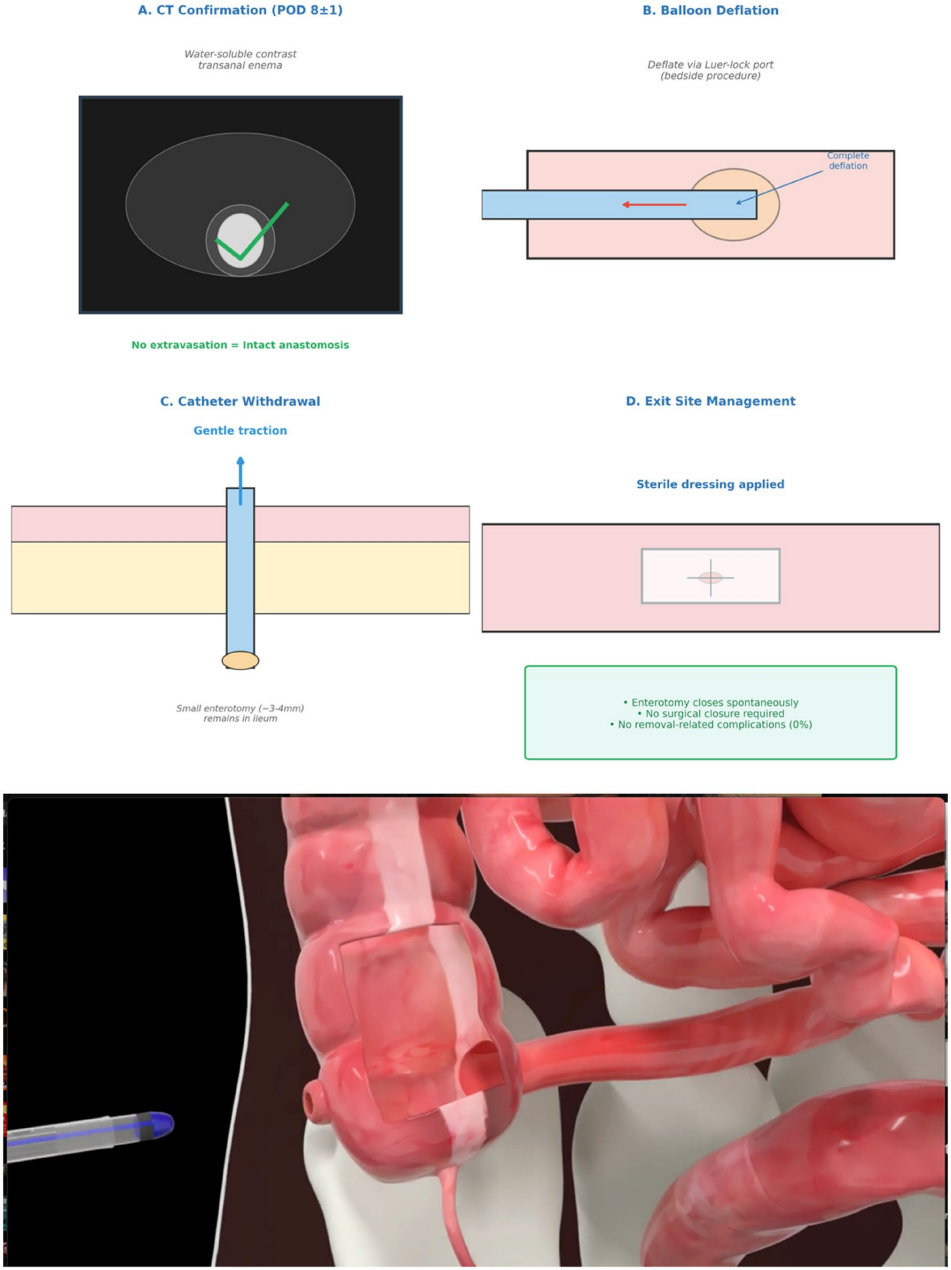
Device removal protocol. **A** CT with trans-anal water-soluble contrast confirming anastomotic integrity at postoperative day 8. **B** Balloon deflation via Luer-lock port. **C** Catheter withdrawal. **D** Exit site with enterotomy (allowed to close spontaneously) and sterile dressing application

Device removal was performed at the bedside in all patients who did not require conversion. No adverse events were associated with the removal procedure itself – specifically, no bleeding, perforation, peritonitis or wound complications occurred during or within 24 h of removal. The small enterotomy site closed spontaneously in all cases without requiring surgical intervention (Fig. 4).

### Metabolic outcomes

No patients developed high-output syndrome. Mean daily effluent output was 485 ± 125 mL (range 200–800 mL), remaining well below the threshold of 1500 mL/day used to define high output. Metabolic stability was maintained in all patients, with no episodes of dehydration requiring intravenous fluid administration, acute kidney injury or clinically significant electrolyte disturbances requiring correction. Among 32 patients with cancer who are scheduled for adjuvant chemotherapy, all successfully initiated treatment within the planned timeframe without delays attributable to diversion-related complications.

### Mortality

One patient (2.3%) died on postoperative day 105. This patient developed left colon ischemia evident at the first postoperative assessment (unrelated to the ALPITube device), required conversion to colostomy and subsequently succumbed to multiorgan failure. The trajectory was attributable to patient-related factors rather than device failure.

### Contextual comparison with literature benchmarks

To provide clinical context, Table 4 presents observed ALPITube outcomes alongside published loop ileostomy benchmarks. These comparisons should be interpreted cautiously as hypothesis-generating observations rather than formal comparative effectiveness analyses, given differences in study design, patient populations and outcome definitions. One-sample binomial tests indicate where observed ALPITube rates fall below literature benchmarks at the *p* < 0.05 level.

**Table 4:** Contextual comparison with published loop ileostomy benchmarks

Outcome	ALPITube *n* (%)	Literature benchmark	*p*-Value^*^
High-output syndrome	0 (0%)	16%	< 0.001
Exit-site/peristomal skin complications	8 (18.6%)	43%	< 0.001
Postoperative ileus	2 (4.7%)	33%	< 0.001
Small bowel obstruction	1 (2.3%)	14%	0.012
Parastomal hernia	0 (0%)	8%	0.028
Stenosis	0 (0%)	16%	< 0.001
Superficial necrosis	0 (0%)	20%	< 0.001
Anastomotic leak	0 (0%)	10%	0.011
Major complications (CD ≥ III)	8 (18.6%)	30%	0.067

These comparisons are presented as contextual information; formal comparative effectiveness requires prospective randomized study *CD* Clavien–Dindo.

^*^One-sample binomial test comparing observed proportion to literature benchmark (one-sided, H_1_: ALPITube below benchmark)

## Discussion

This IDEAL Stage 2a study demonstrates the technical feasibility of ALPITube implantation across two European centres and provides early signals regarding its safety profile and potential advantages over standard loop ileostomy. The device achieved effective anastomotic protection, with no anastomotic leaks in a high-risk population with prevalent neoadjuvant therapy exposure.

### Mechanism of protection and metabolic advantage

The ALPITube achieves anastomotic protection through near-complete faecal stream diversion. The inflated balloon occludes the ileal lumen proximal to the ileocecal valve, channelling intestinal contents through the drainage eyelets – protected by the device cage system against mucosal plugging – into an external collection system. This maintains a low intraluminal pressure environment and reduces mechanical, enzymatic and bacterial stress on the healing anastomosis. The mechanism differs fundamentally from loop ileostomy, where a formal stoma is created through the abdominal wall. The degree of diversion is verified clinically by confirming appropriate effluent output through the catheter and radiologically by CT contrast study before removal. When gravity drainage proves insufficient, the IFU permits low-pressure vacuum-assisted aspiration (5–10 mmHg) via the cage-protected eyelets, offering an additional management tool not available with conventional stomas.

The absence of high-output episodes represents a notable observation. Traditional loop ileostomy disrupts normal intestinal physiology by bypassing the colon’s role in fluid and electrolyte reabsorption. The ALPITube’s controlled-calibre drainage system (10 Fr catheter with limited eyelet size) appears to modulate effluent flow, preventing the unrestricted high-volume losses characteristic of traditional ileostomies. Mean daily output of 485 mL remained well below high-output thresholds, and no patients required intervention for dehydration or electrolyte disturbances. This metabolic stability has cascading implications: reduced risk of acute kidney injury, preserved renal function for nephrotoxic chemotherapy agents and uninterrupted oncological care pathways. All patients with cancer successfully initiated adjuvant treatment without diversion-related delays.

### Conversion rate and learning curve

The 11.6% conversion rate warrants careful interpretation. Conversions occurred due to practical difficulties in maintaining catheter position and function (balloon displacement, peri-catheter leakage, patient intolerance) rather than catastrophic device failures or emergent complications. The temporal pattern – with 80% of conversions occurring in the first 60% of each centre’s experience – is consistent with learning curve effects observed during adoption of new surgical technologies [13, 14]. The specific failure modes identified (device displacement, securement issues, patient discomfort) represent potentially addressable technical challenges through improved device design and refined implantation protocols rather than fundamental limitations of the percutaneous approach.

Importantly, all conversions were performed electively with a low threshold for crossover when day-to-day device function proved suboptimal. This stepwise strategy – attempting ALPITube first with readily available rescue conversion – appears safe and preserves the option for formal diversion when needed. Pre-procedural counselling should explicitly address the possibility of conversion so that patients understand this represents evolution of the diversion strategy rather than treatment failure.

### Device life-cycle safety

A key finding is the absence of removal-related adverse events. Unlike loop ileostomy closure – which requires a second operation under general anaesthesia with morbidity rates of 17–28% – ALPITube removal was performed at the bedside following simple balloon deflation. The small enterotomy site closed spontaneously without surgical intervention. This favourable removal profile supports a complete device life cycle (implantation, maintenance, explantation) with lower aggregate procedural burden than the two-stage ileostomy paradigm.

### Comparison with alternative strategies

ALPITube occupies a distinct position among alternatives to standard loop ileostomy. High-vigilance selective ileostomy protocols (e.g., the Amsterdam approach using C-reactive protein [CRP] monitoring on postoperative days 2–3 combined with selective endoluminal vacuum therapy for detected leaks) require robust surveillance infrastructure and accept that some patients will develop clinical leaks before intervention [9]. Very early closure strategies (within 7 days, as advocated by the Bordeaux group) reduce stoma duration but still require the initial ileostomy construction and a second anaesthetic for early closure [10]. The ghost ileostomy technique exteriorizes a vessel loop around the terminal ileum for potential later activation, but conversion to functioning ileostomy still requires laparotomy [11]. ALPITube provides immediate diversion without formal stoma creation and eliminates the need for any closure procedure in patients who complete the intended device course.

### Limitations

Several limitations require acknowledgment. First, the retrospective design and absence of a concurrent control group preclude causal inferences. The comparisons with literature benchmarks must be interpreted as hypothesis-generating rather than as establishing comparative effectiveness, given potential confounding from differences in patient populations, outcome definitions and follow-up protocols. Second, the sample size of 43 patients limits the precision of effect estimates and power to detect risk factors for complications; multivariable models showed wide confidence intervals and should be considered exploratory. Third, the two-centre design, while improving generalizability over single-centre reports, may still reflect centre-specific practices not representative of broader implementation. Fourth, follow-up was limited to 90 days, and longer-term outcomes including late complications and quality of life remain to be evaluated. Finally, this study does not address cost-effectiveness, an important consideration for health technology assessment.

### IDEAL framework and future directions

This IDEAL Stage 2a study establishes technical feasibility, identifies procedural refinements needed (particularly regarding device securement) and provides preliminary outcome signals. The findings justify progression to IDEAL Stage 2b (Exploration) with prospective data collection using standardized protocols, and ultimately to Stage 3 (Assessment) randomized controlled trials comparing ALPITube with standard loop ileostomy. Future studies should incorporate patient-reported outcomes, quality-of-life measures, cost-effectiveness analysis and longer-term follow-up. Standardization of outcome definitions – particularly for device-specific events – will facilitate cross-study comparisons and meta-analysis.

## Conclusions

ALPITube demonstrates technical feasibility with an acceptable safety profile and signals potential for reduced metabolic and stoma-specific complications compared with standard loop ileostomy. The preservation of metabolic homeostasis and successful adjuvant chemotherapy initiation in all patients with cancer highlights potential oncological advantages. The 11.6% conversion rate reflects early implementation experience likely to improve with technique refinement. The absence of removal-related adverse events supports a favourable complete device life-cycle profile. These IDEAL Stage 2a findings justify progression to prospective comparative studies with adequate power to confirm the clinical benefits and cost-effectiveness of this novel approach to anastomotic protection.

## Supplementary Information

Below is the link to the electronic supplementary material.

Supplementary file1 (DOCX 32 KB)

## Data Availability

The datasets generated during the current study are not publicly available due to patient privacy restrictions under European General Data Protection Regulation (GDPR) regulations, but anonymized data are available from the corresponding author on reasonable request with appropriate ethical approvals.
