# Supplementary figures and images for: Percutaneous ALPITube ileostomy for colorectal anastomotic protection: a multicentre feasibility study
Source: Tech Coloproctol. 2026 May 15;30(1):110. doi: 10.1007/s10151-026-03330-8 (PMC13346299; doi:10.1007/s10151-026-03330-8)

**Procedural video:** <https://www.youtube.com/watch?v=j-Wc-33hdAo>


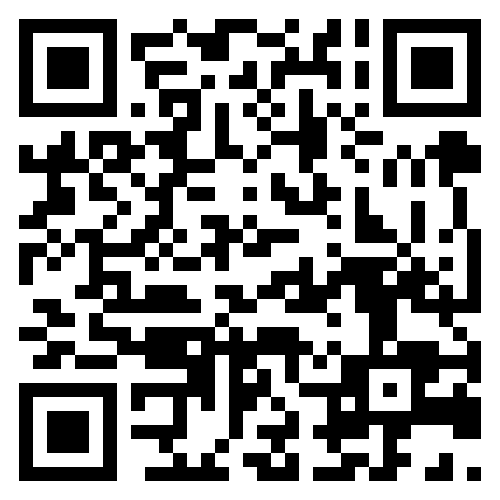

Supplement: Supplementary file 1 — Supplementary file1 (DOCX 32 KB) [file 10151_2026_3330_MOESM1_ESM.docx]
